# Image Representation-Driven Knowledge Distillation for Improved Time-Series Interpretation on Wearable Sensor Data

**DOI:** 10.3390/s25206396

**Published:** 2025-10-16

**Authors:** Jae Chan Jeong, Matthew P. Buman, Pavan Turaga, Eun Som Jeon

**Affiliations:** 1 Department of Computer Science and Engineering, Seoul National University of Science and Technology, Seoul 01811, Republic of Korea; wocks1626@seoultech.ac.kr; 2 College of Health Solutions, Arizona State University, Phoenix, AZ 85004, USA; mbuman@asu.edu; 3 Geometric Media Lab, The GAME School, Arizona State University, Tempe, AZ 85281, USA; pturaga@asu.edu

**Keywords:** time-series data analysis, image representation, persistence image, Gramian angular field, knowledge distillation, wearable sensor data

## Abstract

With the increased demand for wearable sensors, image representations—such as persistence images and Gramian angular fields—transformed from time-series data have been investigated to address challenges in wearables arising from physiological variations, sensor noise, and limitations in capturing contextual information. To preserve the lightweight structural design of models, knowledge distillation (KD) has also been employed alongside image representations during training to distill smaller and more efficient models. Although image representations play a key role in providing richer and more informative features in training a model, their effectiveness within the KD framework has not been thoroughly explored. In this paper, we focus on image representation-driven KD to investigate whether these representations can provide useful knowledge leading to improved time-series interpretation in activity classification tasks. We explore the benefits of integrating image representations into KD, and we analyze the interplay between representation richness and model compactness with different combinations of teacher and student networks. We also introduce diverse KD strategies to utilize image representations, and we demonstrate the strategies with various perspectives, such as analysis of noises, generalizability, and compatibility, across datasets of varying scales to obtain comprehensive and insightful observations. These offer valuable insights for designing efficient and high-performance wearable sensor-based systems.

## 1. Introduction

Wearable sensor data has been widely used in various fields, such as smart home technologies [1], healthcare and medicine for monitoring movement and rehabilitation [2,3], and intelligent surveillance and security to detect abnormal patterns [4]. With this increasing demand, deep learning-based analysis for wearable sensor data has also been actively studied [5,6,7]. Despite these advancements, there are still challenges due to variations between and within individuals, dependency on temporal segmentation strategies and sensor perturbations, and sensitivity to sampling rates [8].

To alleviate these key issues caused by sensor perturbations and individual variations, which hinder the training of deep learning models, image representations (IRs) have been utilized in time-series analysis. The IR is generated by conversion of the raw time-series data, and wearable sensor data have been explored and achieved great successes in improving activity recognition [9,10]. Specifically, persistence images (PIs) and Gramian angular fields (GAFs) are representative methods providing richer contextual information and are increasingly utilized to capture invariant features of time-series data. PIs represent topological characteristics reflecting global and inherent features that are invariant to stretching, bending, and rotation [11,12]. These features can be obtained by Topological Data Analysis (TDA), representing structural variations in the data, and have shown robustness for different types of time-series corruptions [12,13]. On the other hand, the GAF image reflects the similarity between pairs of time points, computed by their angular values in a polar coordinate system [9,14]. This emphasizes temporal correlations and periodic patterns, and it visually represents the periodicity and repetitive patterns of time-series data. Both of these methodologies have commonalities in effectively encoding the structural and temporal characteristics of time series. They enhance pattern recognition and enable more compact and informative feature extraction. These can also be effectively used as standalone representations with deep neural networks. Thus, a model can be trained from scratch using the representation image as an input. Otherwise, the representation can be utilized [14,15]. On the other hand, the IRs can be used as multiple inputs to leverage richer information in model training [9,16]. Even though leveraging IRs aids in improving performance, creating the IRs requires additional time and computational memory, which has to be included in inference. For instance, PIs are generated through TDA; thus, this process has to be included in the testing time to utilize them as an input when the raw time-series data is given alone. When features from PIs are concatenated with the ones from the raw time-series data within a framework, the model size and complexity increase, and additional computing resources are necessarily required, which is not avoidable in the testing time. Therefore, using more input data or stacking more layers is inevitable in general deep learning in training as well as inference time, imposing a critical hindrance in wearable sensor data analysis on devices with constrained computational resources.

To address these problems, knowledge distillation (KD) has been adopted in generating a small model [17]. This technique is a promising one to distill a small model (student model) by leveraging knowledge from a large model (teacher model). Not only a single teacher but also multiple teachers can be utilized in the KD training process to train a robust student model [18]. Since KD can be applied with multimodal teachers, various approaches with the image-based teacher for distilling a student model with time-series data alone have been studied [19,20]. This helps in improving the generalizability of the model and reducing negative effects from perturbations from sensors for activity recognition. Furthermore, additional features from IRs aid in providing useful knowledge that may not be captured by raw time-series features; this, in turn, improves the ability of the model to interpret intrinsic characteristics of time-series data during the training process in KD [18,21]. Even though such methods have demonstrated improvements in generating smaller and more accurate models, there is still limited research analyzing which types of IRs and strategies are effective for distilling a robust model using the KD process on wearable sensor data with varying statistical characteristics, such as window length, complexity, and activity classes.

In this paper, we explore image representation-driven knowledge distillation for human activity recognition on wearable sensor data, which improves time-series data interpretation in training to generate a small model. Firstly, we create IRs for PIs and GAFs, and we investigate the role of these in training a model with learning from scratch and KD. Secondly, we introduce leveraging the representations in diverse KD strategies with single and multiple teachers to distill a single student. This gives light to the interplay between representation richness and model compactness, implying valuable insights for designing efficient and high-performance systems for wearable sensors. We demonstrate the performance on a distilled small model that is implemented with time-series data only and does not require image representations or transformation mechanisms in inference time.

The key highlights and findings of this study are summarized below:

- We introduce the integration of image representations into the KD framework and conduct a comparative analysis between methods that employ image representations and those that do not.
- We perform KD using a single or multiple teachers with different image representations and a diverse capacity of teachers to identify which approach and combination provide the most benefit.
- We clarify the relationships between representation richness and model compactness, providing insights for designing efficient, high-performance wearable-sensor recognition systems.
- We demonstrate the effectiveness of image representation-driven KD strategies in diverse perspectives, including analysis of noises, generalizability, and compatibility with distillation on wearable sensor data. We investigate both small- and large-scale datasets, ensuring consistent and trustworthy observations across varying dataset sizes.

The rest of this paper is structured as follows: In Section 2, we provide an overview of creating image representations from time-series data, as well as KD techniques. In Section 3, we present diverse KD strategies for time-series data analysis with image representations. In Section 4, we show our experimental results and analysis. In Section 5, we provide a comprehensive discussion of the results. Finally, in Section 6, we provide the overall summaries and conclusions.

## 2. Background

This section introduces the theoretical foundations that form the basis of the proposed method. In particular, we focus on three key components: extraction of persistence images through Topological Data Analysis, conversion of Gramian angular field images, and fundamentals of knowledge distillation.

### 2.1. Persistence Image Extraction by Topological Data Analysis

Topological Data Analysis (TDA) is a mathematical framework for extracting hidden and complex topological features from data [11]. The features obtained through TDA are widely used in various tasks to improve performance [16,22,23].

To use TDA with time series, 1D signal data are projected onto an *n*-dimensional point cloud by using a sliding window [24]. Persistent homology is then computed on this point cloud. Points within a given radius ϵ are connected to form a simplicial complex. As the radius increases, the complex grows and topological features (connected components, loops, and voids) appear and disappear, where the time is defined as birth- and death-time, respectively. This multiscale procedure is called a filtration [25]. The resulting birth–death pairs are summarized in a persistence diagram (PD), which is stable under small perturbations of the input [12,26]. However, since PDs are unordered and of variable size, it is difficult to directly apply them to machine learning as well as deep learning. To obtain a fixed-size representation, PDs are converted into persistence images (PIs). To extract PIs, firstly, a persistence surface is built by placing a weighted Gaussian at each point in the PD and summing them. Next, the surface is discretized on a regular grid, and the value of each cell is computed to form a pixel matrix, which is the PI. Higher pixel values indicate higher persistence.

**Figure 1:**
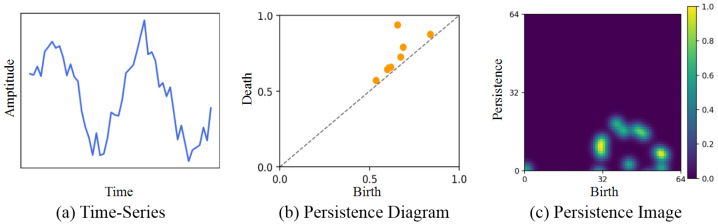
Example time series and its corresponding persistence diagram and persistence image.

With these TDA processes, the obtained PI can provide richer information that complements the original data. However, extracting PIs requires substantial memory and computation and is typically run on CPUs. This causes difficulties in deployment on deep learning models and implementation on small devices. Accordingly, in this paper, TDA is used only on the teacher side to offer additional knowledge that is helpful in distillation; thus, the student does not require TDA in both training and test time. Figure 1 illustrates the example of time-series data and its corresponding PD and PI.

### 2.2. Gramian Angular Field Image Extraction

A Gramian angular field (GAF) transforms time-series data into an image that represents polar coordinates instead of the Cartesian coordinates [27]. The image is a Gramian matrix formed by mapping each value to a polar angle based on cosine summation of angle pairs. The image maintains the time order with its rows and columns, and it encodes relationships between near or distant time steps as diagonal patterns. Also, the main diagonal of this image implies value information of the raw time-series data. Since the Gramian matrix provides additional information that is difficult to obtain with the raw time series alone, many recent studies have incorporated GAFs into deep learning-based methods to improve performance. Specifically, a GAF is able to capture significant differences between similar actions of raw data, which helps in model training for better classification of human activity [9,28,29].

To obtain GAFs in this study, we followed the process explained in the previous study [27] for the generation of GAFs. We explain the steps to obtain a GAF image in detail: First, the raw time series is normalized to the range [−1,1]. Then, each value is mapped to a polar angle as $\phi_{t}=\arccos\left(x_{t}\right)$, where *t* denotes each time step. Next, each entry of the GAF matrix is computed by taking the cosine of the sum of the two angle values:

(1) $${\mathrm{GAF}}_{i,j}=\cos\left(\phi_{i}+\phi_{j}\right),$$

where *i* and *j* are two time steps in the series. Finally, the GAF image is created as follows:

(2) $$\mathrm{GAF}=\left(\begin{array}{cccc} \cos(\phi_{1}+\phi_{1}) & \cos(\phi_{1}+\phi_{2}) & \ldots & \cos(\phi_{1}+\phi_{n})\\ \cos(\phi_{2}+\phi_{1}) & \cos(\phi_{2}+\phi_{2}) & \ldots & \cos(\phi_{2}+\phi_{n})\\ \vdots & \vdots & \ddots & \vdots \\ \cos(\phi_{n}+\phi_{1}) & \cos(\phi_{n}+\phi_{2}) & \ldots & \cos(\phi_{n}+\phi_{n}) \end{array}\right),$$

where *n* is the sequence length, which determines the size of the GAF image. This size can be set to a desired resolution by interpolation or by downsampling the original series. The term $\cos(\phi_{i}+\phi_{j})$ can also be written as a Gramian inner product $<x_{i},x_{j}>$, defined as follows:

(3) $$<x_{i},x_{j}>=x_{i}\cdot x_{j}-\sqrt{1-x_{i}^{2}}\cdot \sqrt{1-x_{j}^{2}}.$$

According to Wang et al. [27], the GAF has two variants: the Gramian Angular Summation Field (GASF), which uses the cosine of the angle sum, and the Gramian Angular Difference Field (GADF), which uses the sine of the angle difference. In the GASF, the main diagonal equals $cos(2\phi_{i})$, so per-time-step magnitude information is retained on the diagonal. In contrast, the GADF has a zero main diagonal by definition, emphasizing directional changes rather than absolute level [14].

**Figure 2:**
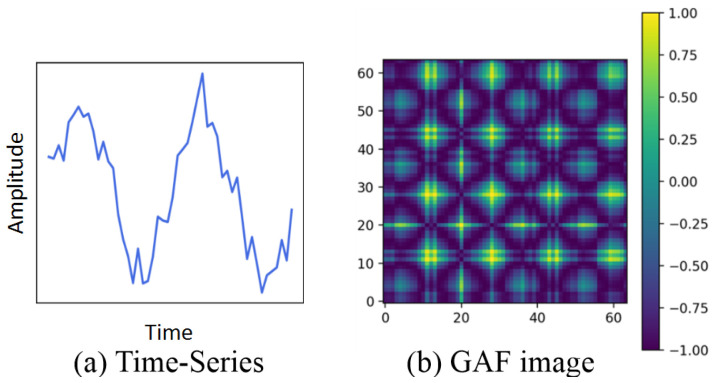
Example of time series and its corresponding GAF image.

Figure 2 depicts the transformation of time-series data into its corresponding GAF representation. Because the GAF calculates the cosine of the sum of angles for each pair (i,j) and (j,i), the generated GAF matrix is symmetric with the main diagonal (with respect to y=x).

Similar to GAFs, there are other texture representations leveraging periodic patterns of time series, one of which is the recurrence plot (RP) [30,31]. RPs include symmetric discretized and continuous patterns, which capture hidden dynamic similarity and anomalies. However, this approach is sensitive to noise and tends to lose knowledge during transformation compared to other representations, such as the GAF. Thus, with these characteristics, GAFs have been more commonly used with deep learning [32,33,34].

### 2.3. Knowledge Distillation

Knowledge distillation (KD) transfers knowledge from a larger teacher model to a smaller student for model compression. This was firstly introduced as a model compression method by Buciluă et al. [35] and was popularized by Hinton et al. [17]. In its standard form, KD aligns the teacher’s and student’s soft output distributions, encouraging the student to approximate the predictive distribution from the teacher. This is also combined with a cross-entropy (CE) loss, mitigating differences between the student’s outputs and the hard labels. The loss function is formed as follows:

(4) $$L_{\mathrm{vanila}\mathrm{KD}}=\left(1-\lambda \right)L_{CE}+\lambda L_{KD},$$

where λ is a hyperparameter in the range [0,1] and balances $L_{\mathrm{CE}}$ and the distillation term $L_{KD}$, $L_{\mathrm{CE}}$ is the cross-entropy loss for the prediction of the student, and the ground truth is defined as follows:

(5) $$L_{CE}=Q\left(\sigma \left(l_{S}\right),y\right)$$

where Q(·) is the cross-entropy function, $l_{S}$ represents the logits of the student, *y* is the ground-truth label, and σ(·) denotes the softmax function. The KD loss $L_{KD}$ is expressed as the scaled Kullback–Leibler divergence between the softened student and teacher logits as follows:

(6) $$L_{KD}=\tau^{2}\;KL\left(\sigma \left(l_{T}/\tau \right),\sigma \left(l_{S}/\tau \right)\right),$$

where $l_{T}$ and $l_{S}$ are the teacher’s and student’s logits, respectively, and τ>1 is the temperature parameter that controls the smoothness of the output distributions.

The standard KD loss is computed solely based on the logits of the student and teacher models. Due to this characteristic, the teacher and student do not necessarily need to share the same input data shape or modality [18,36]. This flexibility allows KD to be effectively applied even when the teacher and student use inputs from different data modalities, enabling the student to gain complementary information from diverse sources. Moreover, multiple teachers, each using different modalities, can be incorporated together in knowledge distillation. In this training process, the KD loss can be formulated as a weighted sum of the individual distillation losses from each teacher:

(7) $$L_{{KD}_{m}}=\left(1-\lambda \right)\;L_{\mathrm{CE}}+\lambda \left[\left(1-\alpha \right)\;L_{{\mathrm{KD}}_{T_{1}}}+\alpha \;L_{{\mathrm{KD}}_{T_{2}}}\right]$$

where α is in the range [0,1] and controls the relative contributions of the first teacher ($T_{1}$) and the second ($T_{2}$), while $L_{{\mathrm{KD}}_{T_{1}}}$ and $L_{{\mathrm{KD}}_{T_{2}}}$ are computed with Equation (6). This is applicable in our experiments utilizing different architectural teachers, where one is trained with time series and the other with IRs.

## 3. Strategies Leveraging Image Representations in KD

**Figure 3:**
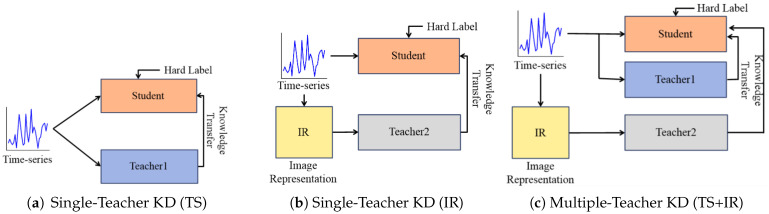
Knowledge distillation strategies for single or multiple teachers utilizing time series (TS) and image representation (IR).

In this section, we introduce different strategies for IR-driven KD, as depicted in Figure 3. KD is usually performed with unimodal data, but it can also be utilized with multimodal data. For instance, in the single-teacher setting, the student takes the raw time series as input, and the teacher can operate on either the raw data or the IR extracted from them. To utilize multimodal data in KD, a multi-teacher approach can be utilized. For this setting, two teachers are used—one on the raw data and the other on the IR—and their knowledge guides the student. More details are provided in the following sections.

### 3.1. Leveraging IR with a Single Teacher

To figure out the effects of IR with a single teacher, here, we set two different strategies with a single teacher in KD. First, we set a vanilla KD that operates with single teacher and the raw data only [17], as shown in Figure 3a. The teacher $T_{1}$ operates on the raw signal with a 1D CNN. When a teacher operates with the same modality of time-series data as a student, the student benefits from the stronger knowledge from a larger model, gaining richer temporal features. Second, to utilize image representation in KD, a teacher trained with IR $T_{2}$ can be used, as shown in Figure 3b. The teacher consists of 2D CNNs. The IR is either a GAF image or a PI. When a teacher performs with a different modality of data from a student, the student obtains complementary supervision by absorbing image-domain knowledge.

With these teachers, a student is trained with a combination of cross-entropy loss on hard labels and a distillation loss from the teacher’s softened logits, as formulated in Equation (4). For both strategies, the student uses 1D CNNs to process the raw time-series input alone. Therefore, the distilled student does not require IR or representation transformation at test time.

### 3.2. Leveraging IR with Multiple Teachers

To use strong knowledge from time series as well as image representation, we set a strategy leveraging multiple teachers trained with different modalities, as depicted in Figure 3c. One teacher $T_{1}$ and a student consist of 1D CNNs and take time series as inputs. On the other hand, the other teacher $T_{2}$ runs with 2D CNNs and performs with IRs created from the raw time-series data. The KD process is mainly the same as the implementation with a single teacher, e.g., both teachers are pre-trained models that are not updated during the training time. Also, even though multiple teachers are leveraged, the distilled student requires only time-series data in both training and test time. As explained in Equation (7), a student mimics both teachers, which implies that the student fuses the knowledge from different teachers. Thus, this training enables the student to obtain the benefits of complementary features. Specifically, the IR provides hidden knowledge that is hardly captured by the raw time-series data. Despite requiring only raw signals at inference, the student can still take advantage of these benefits.

Even though this strategy has advantages, using different modalities in a unimodal framework is difficult, since simple integration may cause confusion and feed knowledge as noises [18]. For example, since the teachers and the student differ in both architecture size and modality, the internal representations can be misaligned, which weakens the distillation. To address this issue, we adopt the annealing strategy (Ann) introduced in the previous study [36], which is mainly used in our experiments for IR-driven KD. For more details on Ann, before distillation begins, we train a vanilla model with the same architecture and capacity as the student from scratch on raw time-series data, using only the cross-entropy loss. The resulting weights are then used to initialize the student, instead of random parameters. This helps to maintain intrinsic features for time series and narrow the representational gap cased by different modalities, thereby stabilizing the early stage of training, and improves the effectiveness of knowledge transfer from the teachers.

## 4. Experiments

### 4.1. Dataset Description and Settings

In this section, we explain the datasets that were used in the evaluations and the settings used to conduct the experiments.

#### 4.1.1. Dataset Description

**GENEActiv:** The GENEActiv dataset [37] was collected by a wearable sensor, consisting of a lightweight, waterproof, and wrist-worn tri-axial accelerometer. The sampling frequency was 100 Hz. With reference to previous studies [36,38], the window size of a sample was set to 500 time steps (5 s) by fully non-overlapped segmentation. The activity classes represent 14 daily activities, such as walking, sitting, and walking on stairs. Each class contains more than 900 samples, and the classes are imbalanced. The training and testing sets consisted of 16k and 6k samples collected by 131 and 43 subjects, respectively. For the subjects, healthy adults aged between 18 and 64 participated. We utilized this dataset with diverse settings, including different window lengths and complexities in classification.

**PAMAP2:** The PAMAP2 dataset [39] is a public dataset consisting of several measurements, such as heart rate, temperature, accelerometers, gyroscopes, and magnetometers. Nine subjects participated, and the original sampling rate was 100 Hz. As in a previous study [40], we used downsampled data at 33 Hz and 100 time steps as the window length, constructed using semi-non-overlapping sliding windows with 78 time steps of overlap [39]. The classes represent 12 different daily activities, such as walking, cycling, and playing soccer. Some subjects have missing data, and the dataset distribution is imbalanced. The evaluation metric for this dataset is leave-one-subject-out, which differs from the one used for GENEActiv.

#### 4.1.2. Experimental Settings

For the extraction of PIs, we followed the processes described in prior work [16] and used the Ripser library to compute persistence diagrams (PDs). The Gaussian kernel parameter in PD computation was set to 0.25 for GENEActiv and 0.015 for PAMAP2. The birth-time ranges for PIs were fixed at [−10,10] and [−1,1] for GENEActiv and PAMAP2, respectively. Each generated PI was normalized by its maximum value and resized to 64×64 pixels. For the GAF images, we used the GASF variant, because the main diagonal keeps each time step’s value, and this has been commonly used for GAFs in deep learning [28,29]. We resized the extracted GAF image to 64×64, the same as the PI resolution, so that they could be processed with the same architecture.

We trained all models by using stochastic gradient descent (SGD) with a momentum of 0.9, a weight decay of $1\times 10^{-4}$, and a batch size of 64. The total number of training epochs was 200. For time-series models on both datasets, the initial learning rate was set to 0.05, decaying by a factor of 0.2 at epoch 10, and subsequently reduced by a factor of 0.1 every $\frac{t}{3}$ epochs, where *t* denotes the total number of epochs. Both the teacher and student networks employed the WideResNet (WRN) architecture [41], which is widely used in knowledge distillation research for performance evaluation [38,42]. Time-series models utilized 1D convolutional layers, whereas image-based models adopted 2D convolutional layers. Regarding hyperparameters, the temperature parameter τ was fixed at 4 for both GENEActiv and PAMAP2, while the balancing weight λ was set to 0.7 and 0.99, respectively, in accordance with the prior studies [19,38]. For both multi-teacher configurations, the default balancing coefficient α was set to 0.7 for GENEActiv and 0.3 for PAMAP2. These values were determined empirically, which is explained further in Section 4.4.1.

For comparisons with various methods of KD, we evaluated diverse knowledge distillation-based methods, including vanilla KD [17], attention transfer (AT) [43], similarity-preserving KD (SP) [44], simple knowledge distillation (SimKD) [45], DIST [46], DCD [47], and feature distillation KD with projector (Projector) (introduced by Miles et al.) [48]. The hyperparameters $\alpha_{AT}$ and $\gamma_{SP}$ were set to 1500 and 1000, respectively. We also compared against multi-teacher approaches such as AVER [49], EBKD [50], CA-MKD [51], Base [36], and Ann [36]. AVER sets the distillation loss weights for both teachers equally (α = 0.5), ensuring an unbiased contribution from each teacher. Base uses the dataset-specific distillation loss by setting α as default values. Ann applies the proposed annealing initialization to the Base configuration. If the prior multi-teacher methods could not be applied with multimodal features, we adopted logits only in their methods for distillation.

### 4.2. Analysis of Different Strategies and Combinations

In this section, we compare the performance of the student models under the different training strategies described in the prior section, i.e., single- and multi-teacher approaches using different modalities (e.g., 1D time series or 2D image representation for GAFs or PIs). The architectural styles and capacities of teacher and student networks importantly affect performance in KD. This has been popularly explored in many previous studies regarding knowledge distillation [38,42]. To investigate how the choice of teacher modalities and architectures influences the effectiveness of knowledge transfer, we organized experiments and comparisons, considering various teacher–student combinations with diverse strategies.

As explained in Table 1, we first presented detailed information for each teacher–student configuration, including the number of parameters and FLOPs for the 1D teacher (Teacher1), the 2D teacher (Teacher2), and the student network, consisting of diverse structures and capacities. Based on these, we computed the compression ratio based on multiple teachers to indicate the compactness of the student compared to the teacher. With this, we evaluated and compared distilled students from various KD methods in terms of classification performance, as discussed in the following sections.

#### 4.2.1. Single and Multiple Teachers

In this section, the architectural configurations of the teacher and student networks summarized in Table 1 are used to analyze the effectiveness of strategies across different scales of datasets. We present the experimental results on three datasets: GENEActiv with 14 classes (in Table 2), GENEActiv with 7 classes (in Table 3), and PAMAP2 (in Table 4).

For results on GENEActiv with 14 classes, as explained in Table 2, for the standard KD with a single teacher among three modalities (TS, PI, and GAF), GAF yields the highest-performing student, and among the multi-teacher approaches, Ann generally outperforms the others. In most of the cases, when the raw time series and GAFs perform together as teachers (TS+GAF), the distilled student achieves higher accuracy than performing with PIs (TS+PI). This shows that GAFs effectively capture the periodic patterns inherent in human activities and provide useful guidance in KD. Furthermore, teachers with architectures closer to that of the student tend to yield better performance than those with much larger capacities. For instance, compared to the results with WRN28-3 teachers, the ones with WRN16-3 teachers distill a better student.

**Figure 4:**
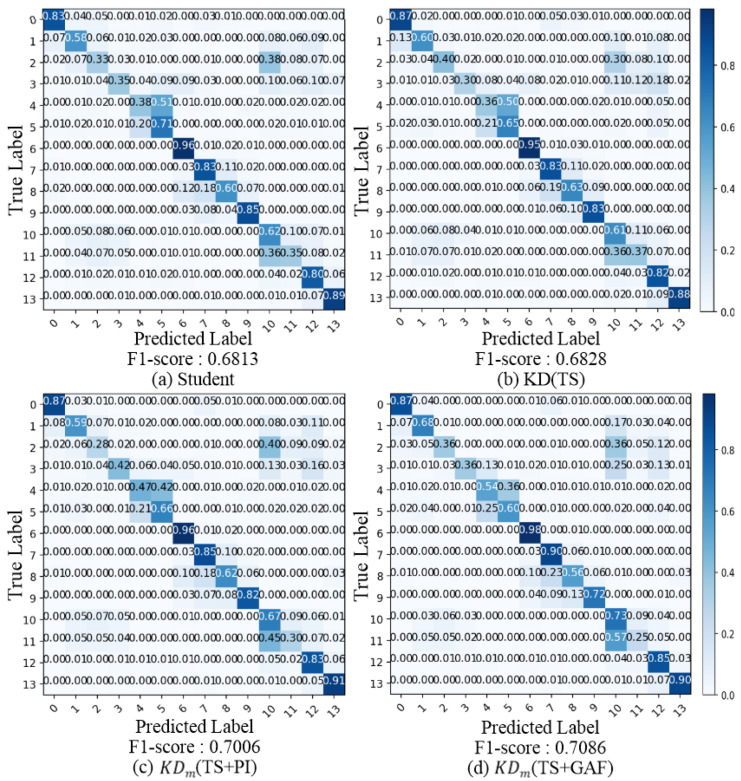
Confusion matrices of students trained with different knowledge distillation methods. All teachers are based on WRN16-3. $KD_{m}$ denotes leveraging multiple teachers for KD, with the teachers indicated in the bracket.

To analyze the effect on class imbalance, Figure 4 presents the confusion matrices and reports F1-scores of students trained under different approaches, such as learning from scratch (Student), a single-teacher-based method (KD(TS)), and multiple-teacher-based methods using IRs ($KD_{m}$(TS+PI) and $KD_{m}$(TS+GAF)). Since the F1-score is calculated as the harmonic mean of precision and recall, it provides a complementary perspective that is particularly informative under imbalanced class distributions. Student and KD(TS) are trained only with time-series data. These models show confusions between class 2 (3 mph treadmill with 5% incline) and class 10 (hard surface walking), between class 4 (6 mph treadmill) and class 5 (6 mph treadmill with 5% incline), and between class 10 (hard surface walking) and class 11 (carpet walking with high heels or dress shoes). These may be caused by similarities of activities, material or environment of clothing, and floor surface.

Compared to Student, KD(TS) improves the overall accuracy, but similar class confusions remain. With PIs added ($KD_{m}$(TS+PI)), the accuracy for class 4 (6 mph treadmill) improves significantly, and the overall diagonal entries become much brighter, indicating better class separability. When the GAF is included ($KD_{m}$(TS+GAF)), the distilled student model achieves the best F1-score. The confusion matrix shows that the accuracy of class 1 (2 mph treadmill) and class 4 (6 mph treadmill) increases further, although that of class 9 (driving a car) shows a slight decrease, whose trend differs from that of Student and KD(TS). However, the result on class 11 is lower, and the class is more confused with class 10 than with the others. These results indicate that each modality is better suited to distinguishing certain classes, and balancing their contributions is crucial in leveraging multimodality.

To explore different window lengths and complexities of tasks, we used a 7-class dataset and evaluated diverse methods and strategies, using the same hyperparameters as in the 14-class experiments. WRN16-1 and WRN16-3 teachers were utilized. Table 3 shows the results. LS denotes a model learned from scratch. ESKD [42] uses an early-stopped teacher, which is used commonly in KD. Full KD uses a teacher resulting from the last epoch of its training. As shown in this table, for this lower-complexity dataset, Ann achieves the highest accuracy among all strategies and KD methods. Additionally, the results show that the overall methods for a window size of 1000 yield higher accuracy compared to implementation on a window size of 500. This indicates that larger amounts of information contained in each sample can provide more attributes to improve the performance.

We also conducted experiments on PAMAP2 with different evaluation metric that is leave-one-subject-out. and containing larger variates. The results are shown in Table 4. For a single teacher, among the standard KD results, the GAF yielded the highest performance among the three modalities. When the teacher and student shared the same architecture (WRN16-1 teacher), the best results were achieved. In contrast, when a PI or time series was the teacher, the highest performance was observed with the WRN16-3 architecture. For multiple-teacher results, Ann achieved the highest or near-highest performance across all modalities and architecture combinations. In particular, the combination of time-series and GAF (TS+GAF) teachers outperformed that of time-series and PI (TS+PI) teachers, although the standalone PI-based teacher showed higher accuracy than the GAF-based teacher. This implies that a GAF provides more effective complementary information to the raw time-series data in KD training. Furthermore, smaller architectural differences between the teacher and the student lead to more effective knowledge transfer and higher overall accuracy, corroborating the findings of previous studies [19,42].

#### 4.2.2. Different Capacities of Teachers

We conducted additional experiments to investigate the effects of architectural differences between teachers in the multi-teacher strategy. In this subsection, we keep the backbone architecture as WideResNet and vary its depth and width to form different teacher configurations. As shown in Table 5, the experiments were performed considering three different conditions: depth-wise, where only the depth of the two teacher models differs; width-wise, where only the width differs; and depth+width-wise, where both depth and width differ simultaneously.

The results show a similar tendency to that explained in Section 4.2.1. Under different architectural combinations of teachers, in all cases, TS+GAF achieves better performance than TS+PI. Also, overall, Ann outperforms Base. For a detailed comparison of the effects on two different IRs, in different depth configurations, TS+PI shows the best results with WRN40-1 Teacher1 and WRN16-1 Teacher2. However, TS+GAF distills the best student with WRN16-1 Teacher1 and WRN40-1 Teacher2, whose teacher models differ from those of TS+PI. For width differences, when WRN16-3 Teacher1 and WRN16-1 Teacher2 are used, TS+PI presents the best results. However, TS+GAF obtains the highest accuracy with WRN16-1 Teacher1 and WRN16-3 Teacher2, which is the opposite configuration to TS+PI. Also, mostly, when Teacher2 is larger than Teacher1, TS+GAF distills a better student. These results representatively show that PIs and GAFs have different effects in distillation, but both improve KD performance to generate better student models, outperforming students that are learned from scratch.

### 4.3. Analysis on Tolerance to Noises

To evaluate the robustness of each method against signal corruption, we conducted experiments by injecting noise into the testing set. Two types of perturbations were considered—continuous missing segments, and Gaussian noise—both of which have been widely used in prior studies for assessing the resilience of time-series models [52,53]. These corruptions were applied simultaneously, and their severity was controlled through parameterized settings that specified the proportion of values to be removed and the standard deviation of the Gaussian perturbation. Based on these parameters, we defined three corruption levels—Level 1 (0.15, 0.06), Level 2 (0.22, 0.09), and Level 3 (0.30, 0.12)—with higher levels corresponding to stronger noise intensities. It is important to note that all models were trained on the original clean dataset, and the corruptions were introduced only during testing.

**Figure 5:**
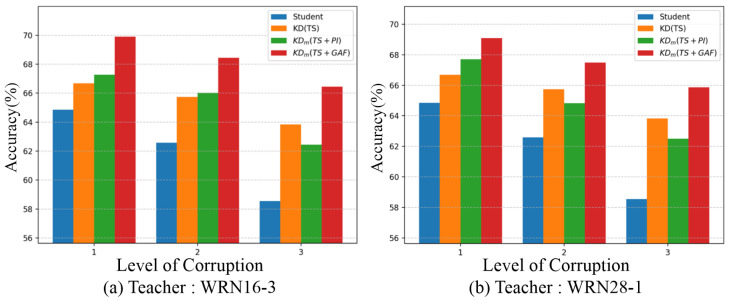
Accuracy (%) on noisy testing sets under different knowledge distillation methods. All teachers are based on (**a**) WRN16-3 and (**b**) WRN28-1. $KD_{m}$ indicates Ann trained by IR guidance in KD with multiple teachers.

Figure 5a shows that the student trained from scratch achieved higher accuracy than KD(TS) across all corruption levels. In contrast, Figure 5b shows that KD(TS) outperformed Student, suggesting that when the teacher and student share the same width, signal-based distillation can provide more improved robustness.

For both teachers, IR-driven KD with multiple teachers exhibited much smaller accuracy degradation under noise compared to models learned with a single modality (Student and KD(TS)), indicating that IR improves the ability of tolerance to noises throughout the KD process. In Figure 5a, KD(TS) is consistently lower than $KD_{m}\left(\mathrm{TS}+\mathrm{PI}\right)$. However, in Figure 5b, KD(TS) maintains higher performance than $KD_{m}\left(\mathrm{TS}+\mathrm{PI}\right)$ except at Level 1. In contrast, $KD_{m}\left(\mathrm{TS}+\mathrm{GAF}\right)$ shows the best performance in all cases. These results show that GAFs help better understanding of intrinsic features than PIs within KD in the previous study [36]. Thus, GAF representations provide more robust complementary information under perturbations.

### 4.4. Analysis of Sensitivity and Compatibility

We conducted additional experiments to investigate both the sensitivity of different KD strategies and their compatibility with other KD techniques. In this section, we analyze the effect of the hyperparameter α, which controls the weighting between the two teacher losses in the multiple-teacher-based KD strategy. We also employ parametric plots to further investigate the relationships between different KD strategies. In addition, we use t-SNE visualizations to explore how different KD methods shape the feature space learned by the student. Finally, we evaluate how IR-driven KD methods perform when combined with prior KD approaches such as AT and SP.

#### 4.4.1. Sensitivity Analysis

**Hyperparameters:** We conducted experiments to evaluate the sensitivity of the multi-teacher setting to the hyperparameter α, which controls the relative contribution of each teacher in the multiple-teacher-based distillation loss.

**Figure 6:**
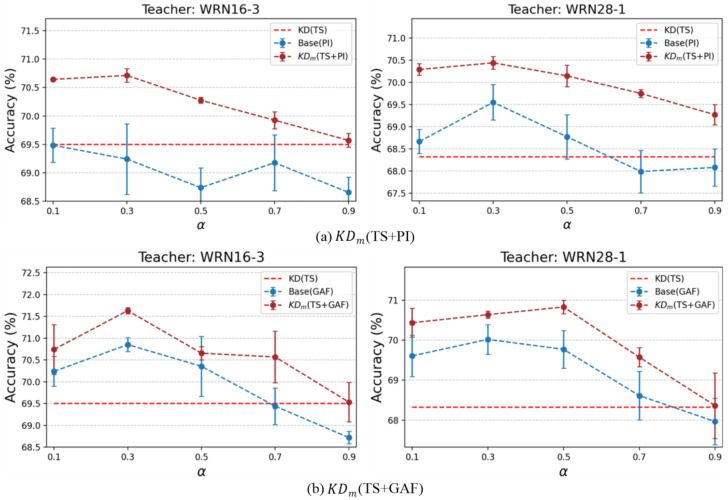
Accuracy (%) of the student model (WRN16-1) depending on the hyperparameter α in the multi-teacher setting on GENEActiv. Both Base and $KD_{m}$ are trained by IR guidance in KD with multiple teachers. $KD_{m}$ indicates Ann.

Figure 6a illustrates the results on α with Base and $KD_{m}\left(\mathrm{TS}+\mathrm{PI}\right)$ with WRN16-3 and WRN28-1 as teachers. When the Ann strategy was applied ($KD_{m}$), both teacher settings achieved their highest accuracy at α=0.3. For Base, the student achieved its highest performance at α=0.3 when the teacher was WRN28-1. On the other hand, with WRN16-3 as the teacher, the highest accuracy was observed at α=0.1, and α=0.3 provided the second-best result.

Figure 6b shows the case of $KD_{m}\left(\mathrm{TS}+\mathrm{GAF}\right)$. For Base, the best performance was consistently obtained at α=0.3. For Ann ($KD_{m}$), the highest accuracy was achieved at α=0.3 with WRN16-3 as the teacher, and at α=0.5 with WRN28-1 as the teacher. Based on these results, we set α=0.3 for GENEActiv in both $KD_{m}\left(\mathrm{TS}+\mathrm{PI}\right)$ and $KD_{m}\left(\mathrm{TS}+\mathrm{GAF}\right)$, where the α generated the best performance overall. For PAMAP2, we chose α=0.7 empirically.

**Parametric Plot:** To further analyze the impact of knowledge distillation strategies, we employed parametric plots, which illustrate the behavior of the model when two independently trained solutions are interpolated [54,55]. We used two sets of model parameters obtained from independently trained students. Referring to previous studies [54,55], the interpolated model was defined as $\psi \left(\left(1-\eta \right)x_{a}+\eta x_{b}\right)$, where η∈[−1,2] controls the interpolation ratio and $x_{a},x_{b}$ are the parameters of the two solutions. Using this formulation, parametric plots can reveal the stability and similarity of the solutions under different interpolation ratios.

**Figure 7:**
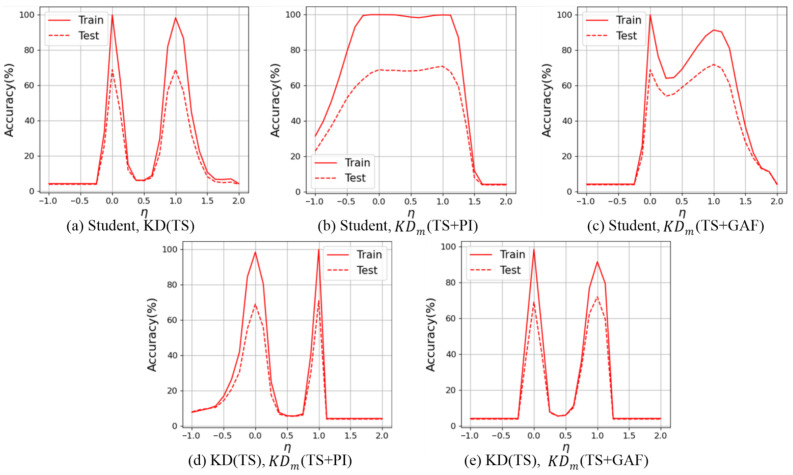
Parametric plots of students (WRN16-1) trained with different KD strategies, with teachers fixed as WRN16-3. $KD_{m}$ denotes KD training with an annealing strategy and multiple teachers, including TS and IR.

In Figure 7a, η=0 and η=1 correspond to the cases where the parameters of only one model are used. At both endpoints, the training accuracy reaches nearly 100%, while the gap between training and testing accuracy is about 30%. When η lies between 0 and 1, the accuracy gradually decreases, and it drops to below 10% around η=0.5. This sharp decline indicates that the independently trained model parameters represent substantially different solutions, leading to severe misalignment when interpolated.

In Figure 7b, the student trained with $KD_{m}$(TS+PI) and Ann is compared against the student learned from scratch with time series only. Since the student distilled by $KD_{m}$ and Ann is initialized with the same weights as the baseline student trained from scratch, the interpolation between η=0 and η=1 maintains relatively higher accuracy compared to Figure 7a. This implies that both students possess similar weights, and that $KD_{m}$ enables a student model to preserve weights that are helpful for understanding the original time series. In Figure 7c, there is a comparison between Student and the student distilled by $KD_{m}$ (TS+GAF). While the interpolation still preserves a reasonable level of accuracy between η=0 and η=1, the performance in the intermediate region drops further than that in Figure 7b. This suggests that the parameters of the student trained with $KD_{m}$(TS+GAF) diverge more noticeably from those of the baseline student. Moreover, unlike in panels (a) and (b), where the training accuracy of both models approaches 100%, the student trained with $KD_{m}$(TS+GAF) only reaches about 90% training accuracy. However, $KD_{m}$(TS+GAF) outperforms the others in testing accuracy. Also, for η>1, the testing accuracy drops more gently than that depicted in panel (b). This indicates that GAFs may add more conflicting features to the raw time-series features in training compared to PIs. However, this ultimately turns out to improve generalizability, a tendency that is commonly observed in schemes of augmentation strategies.

Figure 7d,e show interpolations comparing KD(TS) with $KD_{m}$(TS+PI) and $KD_{m}$(TS+GAF), respectively. In both cases, the accuracy between η=0 and η=1 drops sharply, which implies that the weights obtained for KD(TS) differs from that obtained for Student.

Also, the solutions obtained by the students trained with $KD_{m}$(TS+PI) and $KD_{m}$(TS+PI) differ substantially from that obtained by the student trained with KD(TS). Furthermore, this indicates that training with IR based on multiple teachers and Ann within KD aids in not only leveraging richer contextual knowledge but also posing intrinsic time-series features to distill a robust student.

**Figure 8:**
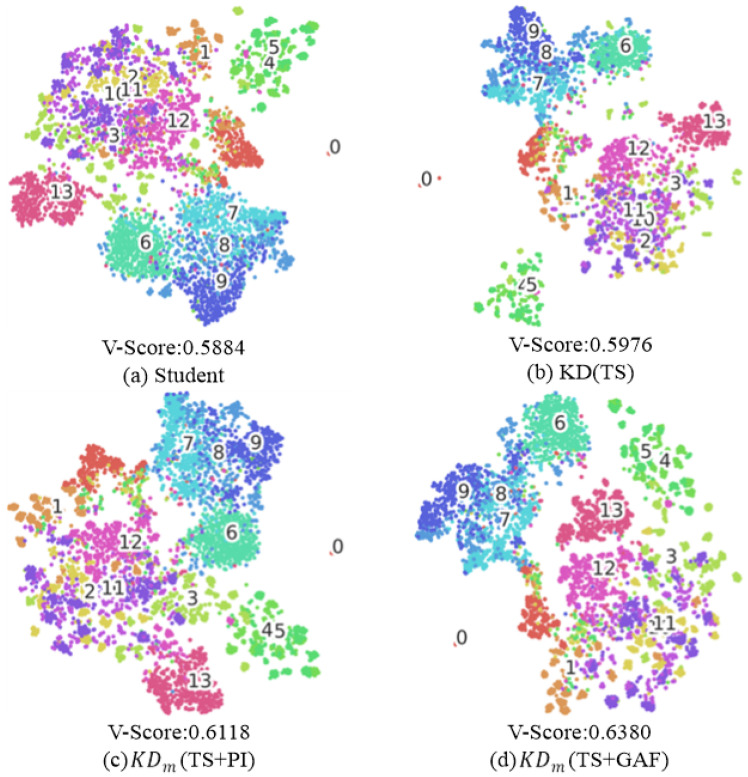
The t-SNE plots and V-Scores of students obtained from different knowledge distillation strategies. Each student is trained with WRN16-1, and the teachers are fixed as WRN16-3.

**T-SNE Plot:** We examined the t-SNE plots and V-Scores of student models trained with different methods to evaluate the effects of each approach on completeness and homogeneity in clustering [56]. Figure 8a shows the student model trained from scratch, where significant confusion can be observed among classes 2, 10, and 11, as well as between classes 4 and 5. Figure 8b, which depicts the student trained with KD(TS), also shows a similar confusion pattern, although there is a slight improvement, as demonstrated by the V-Score. Figure 8c presents the results of the student distilled by $KD_{m}$ with PI guidance through KD, where the V-Score increases to 0.6118. Unlike Figure 8a,b, classes 2 and 12 are more clearly separated, and the clustering distance between classes 4 and 5 increases. Figure 8d illustrates the student distilled by $KD_{m}$ with GAF guidance through KD, which achieves the highest V-Score of 0.6380. In this case, the cluster between classes 4 and 5 becomes more distinct, and classes 11 and 12 are more separated. These results show that incorporating additional image modalities in KD helps achieve higher V-Scores compared to using time series alone, which indicates the robustness of IR guidance in KD training to distill a superior student.

#### 4.4.2. Distillation Compatibility

Although our previous experiments considered only logit-based distillation, KD can also be applied in the feature space. Representative methods include attention transfer (AT) [43] and similarity-preserving KD (SP) [44]. Accordingly, to investigate the compatibility of different strategies with feature-based KD methods, we conducted experiments by applying these methods (AT and SP) to multi-teacher-based KD with IR. In all experiments, to maintain the original processes introduced from prior studies [43,44], we applied AT and SP only to the time-series-trained teacher branch as auxiliary losses. We used two different teacher capacities: WRN28-1 and WRN28-3.

**Figure 9:**
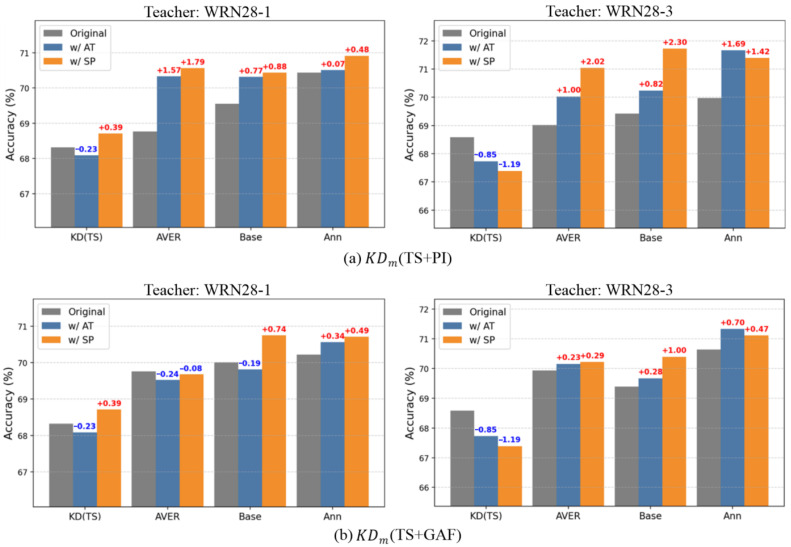
Effects of AT and SP on multi-teacher knowledge distillation relative to the original for TS+PI and TS+GAF. Teachers are WRN28-1 and WRN28-3.

Figure 9 reports the results. For WRN28-1 teachers (TS and PI) in Figure 9a, adding AT or SP improves accuracy for AVER, Base, and Ann. SP provides slightly larger gains than AT in all cases. AVER outperforms Base, which suggests that the optimal α can shift when auxiliary features are introduced in the KD process. For WRN28-3 teachers, we observed a dissimilar trend compared to the WRN28-1 teachers. KD(TS) does not perform well when combined with AT and SP. In contrast, all IR-driven KD methods show significant improvements. More specifically, in Table 2, a teacher much larger than the student appeared to provide limited benefit, whereas in this figure, the student maintains strong performance despite the capacity gap between the teacher and student. This effect may be because AT and SP feed richer information and provide more bridging to decrease gaps between teacher and student; thus, larger capacity of teacher or different teacher modalities can also provide knowledge more effectively.

For the WRN28-1 TS and GAF teachers in Figure 9b, AVER and Base show degraded performance, whereas Ann yields improvements with both AT and SP. Also, SP generally outperforms AT. The results with WRN28-3 teachers definitely show the robustness of IR-driven KD, which is the same as in panel (a). Interestingly, when TS+PI is implemented with other KD methods, the distilled student performs better than that distilled by TS+GAF, which is much different from the previous results provided in Section 4.2.1. This implies that PIs and GAFs provide different guidance in KD and possess different compatibilities.

Overall, these results indicate that IR-driven multiple-teacher-based KD is compatible with AT and SP to generate synergistic effects in providing useful knowledge to distill a strong student. Here, we simply used the hyperparameters $\alpha_{AT}$ and $\gamma_{SP}$, which were empirically determined by unit testing. Thus, there is more potential that the better student can be distilled by selecting optimal hyperparameters considering the interactions between diverse strategies.

### 4.5. Processing Time

We evaluated the processing time of various methods on the testing set of GENEActiv. This testing set contained approximately 6k samples, and the batch size was fixed to 1. All experiments were conducted on a desktop equipped with an AMD Ryzen Threadripper PRO 5955WX (16 cores/32 threads, base clock 3.6 GHz), 256 GB memory, and an NVIDIA GeForce RTX 4090 with 24 GB memory. Table 6 reports both the GPU and CPU processing times required to train a model learned from scratch (teacher model), as well as the accuracy and processing time of the corresponding students trained with different methods. Since PIs could only be generated on the CPU, we report the PI generation time on the CPU, which is not avoidable in inference time. Similarly, GAF images were generated on the CPU, and their generation time was also included in the testing time.

For the time-series teacher, the processing time was 29.63 s on the CPU and 22.23 s on the GPU, showing faster training on the GPU. For teachers using PIs and GAFs (WRN16-3), the processing time was approximately 29 s on the CPU and 16 s on the GPU; however, they could not avoid the IR generation time in the testing phase. Students distilled by KD-based methods (KD and $KD_{m}$) required only 13.57 s on the CPU and 15.03 s on the GPU. Despite the much shorter processing time compared to models learned from scratch, they achieved higher performance. Specifically the models with $KD_{m}$ incorporating knowledge from multiple teachers showed the best accuracies on the classification task. The results show that the CPU is faster than the GPU. This is because GPU utilization is limited by memory transfer overhead and small batch sizes, which hinder the benefits of GPU parallelism, especially with small-model implementation [57,58]. This indicates that the student model that we chose is definitely small (0.06 M parameters) and well designed for our purpose. Thus, with the proposed method, a compact model can be developed, which can perform on small devices with constrained power and computational resources.

## 5. Discussion

Compared to using a single time-series modality, image representation-driven KD required a longer training time, yet the distilled students achieved higher performance. Through the reported results, we found that transforming time series to image representations reveals complementary information that is not easily captured by the raw time series alone.

GENEActiv includes similar activities (e.g., walking) but different speeds, and it has imbalanced distributions, which causes more confusion in model training. The confusion matrices in Figure 4 clearly show a class-dependent effect of diverse KD strategies on GENEActiv. IR-trained teachers improve the overall classification performance but cause slight degradations between some classes. This suggests that a single fixed weight has some limitations in reflecting diverse variations in the patterns of interaction between samples and teachers. Thus, using an adaptive α may provide more optimal weights on each sample and better results. Such adaptivity would consume more resources, but it could deliver higher performance.

Additionally, we observed that IR may interfere with straightforward feeding of features to the student in the training phase. Despite this, the approach enhances the overall effectiveness of KD and particularly aids in interpreting hidden time-series features, as demonstrated by diverse evaluations with much higher accuracy and generalizability compared to other methods. Table 5 further shows that architectural differences between teachers, such as variations in depth or width, did not hinder knowledge transfer; that is, heterogeneous teacher–student combinations sometimes yielded higher accuracy than identical ones. Notably, the parametric plots in Figure 7 provide an interesting observation: the student trained from scratch has largely different optimized weight parameters compared to the model distilled by KD using time-series data. In contrast, the model distilled by IR-driven KD has more similar parameters to those of the student learned from scratch. This prominently indicates that an annealing strategy used in multiple-teacher-based KD enables image representations to serve as complementary information. Also, as reported in our experiments, the image representations of both PIs and GAFs contributed to improving KD, but their roles were not the same. As shown in Figure 5, GAFs showed higher noise tolerance than other methods, implying that GAFs are useful in the preservation of intrinsic temporal information, which is mapped along the main diagonal in their GASF form, acting as a strength under corrupted signals. On the other hand, Figure 9 shows that PIs achieved larger accuracy gains, indicating that their ability to capture topological features of the global structure made them more compatible with additional distillation methods such as AT and SP.

Beyond these findings, since most of the experiments used WideResNet variants, evaluating cross-architecture distillation (e.g., Resnet to VGG or WRN to MobileNet) could be assessed for our extended experiments.

## 6. Conclusions

In this study, we investigated image representation-driven KD for wearable sensor data analysis. IR-driven KD consistently outperformed single-teacher-based KD strategies. We explored the performance of KD approaches with different architectural and capacity combinations for teacher and student on various scales and complex datasets, which are crucial in KD processes. Also, for further rigorous evaluations, we analyzed the effectiveness from different perspectives, such as tolerance to corruption, sensitivity analysis, parametric plots, and compatibility. Overall, training a student with GAFs achieved better accuracy than with PIs. However, when other distillation methods (e.g., AT and SP) were utilized simultaneously, KD with PIs tended to achieve better performance, implying better compatibility and improved generalizability in feature space. Furthermore, IR-driven multi-teacher KD is a practical approach, since it generates a small model implementing time-series data alone in inference. With our findings, we can conclude that IR-driven KD aids in better interpreting time series in distillation, thereby distilling a superior student. We also expect that these analyses could provide insights in developing wearable sensor-based systems with various applications.

In future work, we will aim to build a stronger student by transferring combined and synergistic features through multi-teacher models trained by PIs, GAFs, and time series. We would like to investigate the interaction between IRs and develop a means to adopt adaptive weighting between teachers. In addition, we will extend the logit-based distillation to incorporate feature-based methods to improve performance.

## Figures and Tables

**Table 1:** Description of teacher and student networks. The compression ratio was computed with two teachers. WideResNet is indicated with WRN(depth)-(channel multiplication). TS denotes time series.

DB	Teachers	Student	FLOPs			# of Params			Compression Ratio
			Teacher1	Teacher2	Student	Teacher1	Teacher2	Student	
			(TS)	(PI/GAF)	(TS)	(TS)	(PI/GAF)	(TS)	
GENEActiv	WRN16-1	WRN16-1	11.03 M	108.97 M	11.03 M	0.06 M	0.18 M	0.06 M	25.93%
	WRN16-3		93.95 M	898.52 M		0.54 M	1.55 M		2.94%
	WRN28-1		22.22 M	224.28 M		0.13 M	0.37 M		12.36%
	WRN28-3		192.01 M	1923.93 M		1.12 M	3.29 M		1.39%
PAMAP2	WRN16-1	WRN16-1	2.39 M	131.02 M	2.39 M	0.06 M	0.18 M	0.06 M	25.88%
	WRN16-3		19.00 M	921.03 M		0.54 M	1.56 M		3.01%
	WRN28-1		4.64 M	246.56 M		0.13 M	0.37 M		12.52%
	WRN28-3		38.64 M	1947.13 M		1.12 M	3.30 M		1.43%

**Table 2:** Comparison of various knowledge distillation methods with different strategies in terms of classification accuracy (%) on GENEActiv. **Bold** and red indicate the best and second-best results, respectively.

Model	Teacher1		WRN16-1	WRN16-3	WRN28-1	WRN28-3
	(Time Series)		(67.66)	(68.89)	(68.63)	(69.23)
	Teacher2		WRN16-1	WRN16-3	WRN28-1	WRN28-3
	(PI)		(58.64)	(59.80)	(59.45)	(59.69)
	(GAF)		(63.32)	(64.03)	(64.00)	(65.39)
	Student		WRN16-1			
	(Time Series)		(67.66 ± 0.45)			
Single Teacher	GAF	KD	70.32	70.37	69.52	69.77
			±0.07	±0.03	±0.09	±0.09
	PI	KD	67.83	68.76	68.51	68.46
			±0.17	±0.73	±0.01	±0.28
	Time Series	KD	69.71	69.50	68.32	68.58
			±0.38	±0.10	±0.63	±0.66
		AT	68.21	69.79	68.09	67.73
			±0.64	±0.36	±0.24	±0.27
		SP	67.20	67.85	68.71	67.39
			±0.36	±0.24	±0.46	±0.49
		SimKD	69.39	69.89	68.92	68.80
			±0.18	±0.11	±0.40	±0.38
		DIST	68.20	69.71	69.23	68.18
			±0.28	±0.15	±0.19	±0.60
		Projector	69.64	70.28	69.43	69.09
			±0.53	±0.38	±0.47	±0.38
		DCD	70.56	70.17	70.05	69.22
			±0.12	±0.2	±0.41	±0.35
Multiple Teachers	TS + PI	AVER	68.99	68.74	68.77	69.02
			±0.76	±0.35	±0.70	±0.50
		EBKD	68.43	69.24	68.45	67.50
			±0.25	±0.25	±0.73	±0.40
		CA-MKD	69.33	69.80	69.61	68.81
			±0.61	±0.16	±0.57	±0.79
		Base	69.09	69.24	69.55	69.42
			±0.37	±0.62	±0.41	±0.58
		Ann	70.15	70.71	70.44	69.97
			±0.03	±0.12	±0.10	±0.06
	TS + GAF	AVER	69.93	70.35	69.76	69.93
			±0.76	±0.35	±0.70	±0.50
		EBKD	68.85	67.84	68.51	68.25
			±0.41	±0.20	±0.67	±0.68
		CA-MKD	70.48	69.98	69.64	70.01
			±0.86	±0.32	±0.56	±0.25
		Base	70.39	70.85	70.01	69.39
			±0.45	±0.16	±0.37	±0.13
		Ann	**70.88**	**71.63**	**70.63**	**70.64**
			±0.13	±0.24	±0.35	±0.40

**Table 3:** Comparison of various knowledge distillation methods in terms of classification accuracy (%) on GENEActiv with 7 classes and different window lengths. **Bold** and red indicate the best and second-best results, respectively.

Method			Window Length			
			1000		500	
LS		WRN16-1	89.29 ± 0.32		86.83 ± 0.15	
		WRN16-3	89.53 ± 0.15		87.95 ± 0.25	
		WRN16-8	89.31 ± 0.21		87.29 ± 0.17	
KD Method			Teacher Model			
			WRN16-1	WRN16-3	WRN16-1	WRN16-3
Single Teacher	Time Series	ESKD	89.79 ± 0.32	89.88 ± 0.07	87.44 ± 0.53	88.16 ± 0.15
		Full KD	88.78 ± 0.72	89.84 ± 0.21	86.28 ± 1.02	87.05 ± 0.19
		AT	90.10 ± 0.49	90.32 ± 0.09	87.25 ± 0.22	87.60 ± 0.22
		SP	87.08 ± 0.56	88.47 ± 0.19	87.65 ± 0.11	87.69 ± 0.18
		SimKD	90.25 ± 0.22	90.47 ± 0.32	87.24 ± 0.09	88.16 ± 0.37
		DIST	90.18 ± 0.31	90.20 ± 0.39	87.62 ± 0.02	87.05 ± 0.31
		DCD	89.72 ± 0.91	90.20 ± 0.39	87.82 ± 0.80	88.06 ± 0.75
		Projector	90.01 ± 0.70	90.23 ± 0.36	87.75 ± 0.90	87.93 ± 0.72
Multiple Teachers	TS + PI	AVER	90.01 ± 0.46	90.06 ± 0.33	87.53 ± 0.16	87.05 ± 0.37
		EBKD	90.35 ± 0.12	89.82 ± 0.14	87.51 ± 0.41	87.66 ± 0.28
		CA-MKD	90.01 ± 0.28	90.13 ± 0.34	87.14 ± 0.25	88.04 ± 0.26
		Base	90.22 ± 0.73	89.98 ± 0.16	88.52 ± 0.68	87.85 ± 0.51
		Ann	90.44 ± 0.16	90.71 ± 0.15	88.18 ± 0.12	88.26 ± 0.24
	TS + GAF	AVER	90.32 ± 0.94	90.18 ± 0.83	88.44 ± 0.03	88.13 ± 0.76
		EBKD	89.55 ± 0.52	89.91 ± 1.04	87.91 ± 0.18	88.07 ± 0.52
		CA-MKD	90.25 ± 1.06	91.28 ± 0.58	87.74 ± 0.44	88.00 ± 0.25
		Base	90.44 ± 0.18	90.58 ± 0.11	88.31 ± 0.71	88.71 ± 0.24
		Ann	**91.14** ± 0.12	**91.84** ± 0.24	**88.79** ± 0.20	**88.93** ± 0.39

**Table 4:** Comparison of various knowledge distillation methods and different strategies in terms of classification accuracy (%) on PAMAP2. **Bold** indicates the best results.

Model	Teacher1		WRN16-1	WRN16-3	WRN28-1	WRN28-3
	(Time Series)		(85.27)	(85.80)	(84.81)	(84.46)
	Teacher2		WRN16-1	WRN16-3	WRN28-1	WRN28-3
	(PI)		(86.93)	(87.23)	(87.45)	(87.88)
	(GAF)		(81.44)	(82.29)	(81.90)	(82.98)
	Student		WRN16-1			
	(Time Series)		(82.99±2.50)			
Single Teacher	GAF	KD	87.57	85.03	85.57	85.42
			±2.06	±2.34	±2.58	±2.43
	PI	KD	85.04	86.68	85.08	85.39
			±2.58	±2.19	±2.44	±2.35
	TS	KD	85.96	86.50	84.92	86.26
			±2.19	±2.21	±2.45	±2.40
		DCD	85.58	85.29	83.91	85.69
			±2.29	±2.45	±2.56	±2.56
		Projector	86.61	85.07	84.59	**86.78**
			±2.04	±2.32	±2.53	±2.26
Multiple Teachers	TS + PI	Base	85.91	86.18	85.54	86.04
			±2.32	±2.37	±2.26	±2.24
		Ann	86.09	87.12	85.89	86.33
			±2.33	±2.26	±2.26	±2.30
	TS + GAF	Base	87.22	86.56	84.66	86.41
			±2.23	±2.21	±2.51	±2.32
		Ann	**88.69**	**87.57**	**86.58**	86.77
			±1.83	±2.08	±2.33	±2.31

**Table 5:** Accuracy (%) with different structures of teachers on GENEActiv. Green denotes improvement compared to a model learned from scratch (Student). **Bold** indicates the best results.

Method		Architecture Difference											
		Depth				Width				Depth + Width			
Teacher1 (Time Series)		WRN	WRN	WRN	WRN	WRN	WRN	WRN	WRN	WRN	WRN	WRN	WRN
		16-1	16-1	28-1	40-1	16-1	16-3	28-1	28-3	28-1	28-3	40-1	16-1
		0.06M	0.06M	0.1M	0.2M	0.06M	0.5M	0.1M	1.1M	0.1M	1.1M	0.2M	0.06M
		(67.66)	(67.66)	(68.63)	(69.05)	(67.66)	(68.89)	(68.63)	(69.23)	(68.63)	(69.23)	(69.05)	(67.66)
Teacher2		WRN	WRN	WRN	WRN	WRN	WRN	WRN	WRN	WRN	WRN	WRN	WRN
		28-1	40-1	16-1	16-1	16-3	16-1	28-3	28-1	16-3	40-1	28-3	28-3
		0.1M	0.6M	0.2M	0.2M	1.6M	0.2M	3.3M	0.4M	1.6M	0.6M	3.3M	3.3M
(PI)		(59.45)	(59.67)	(58.64)	(58.64)	(59.80)	(58.64)	(59.69)	(59.45)	(59.80)	(59.67)	(59.69)	(59.69)
(GAF)		(64.00)	(64.35)	(63.32)	(63.32)	(64.03)	(63.32)	(65.39)	(64.00)	(64.03)	(64.35)	(65.39)	(65.39)
Student (Time Series)		WRN16-1											
		0.06M ( 67.66±0.45)											
TS + PI	Base	68.71	68.41	67.89	68.33	68.77	68.92	68.26	69.09	68.04	68.29	68.90	68.15
		±0.36	±0.27	±0.27	±0.17	±0.43	±0.79	±0.13	±0.59	±0.24	±0.27	±0.50	±0.23
	Ann	69.95	69.86	70.34	70.56	69.68	71.06	70.28	69.95	70.28	69.87	70.49	69.65
		±0.05	±0.07	±0.14	±0.04	±0.14	±0.02	±0.08	±0.07	±0.13	±0.23	±0.05	±0.04
		(2.29)	(2.20)	(2.68)	(2.90)	(2.02)	(3.40)	(2.62)	(2.29)	(2.62)	(2.21)	(2.83)	(1.99)
TS + GAF	Base	70.57	70.18	69.48	70.37	70.58	**71.07**	70.02	69.86	69.68	69.69	70.19	69.97
		±0.52	±0.76	±0.19	±0.18	±0.49	±0.36	±0.10	±0.68	±0.38	±0.58	±0.4	±0.49
	Ann	**70.90**	**71.53**	**70.48**	**70.83**	**71.32**	70.92	**70.42**	**70.87**	**70.57**	**70.43**	**71.39**	**70.99**
		±0.30	±0.68	±0.43	±0.25	±0.14	±0.22	±0.22	±0.74	±0.60	±0.56	±0.50	±0.71
		(3.24)	(3.87)	(2.82)	(3.17)	(3.66)	(3.26)	(2.76)	(3.21)	(2.91)	(2.77)	(3.73)	(3.33)

**Table 6:** Accuracy and processing time of various models on GENEActiv.

Model	Learning from Scratch			KD			${\mathrm{KD}}_{m}$	
	TS (1D)	PImage (2D)	GAF Image (2D)	TS	PI	GAF	${\mathrm{KD}}_{m}$ (TS+PI)	${\mathrm{KD}}_{m}$ (TS+GAF)
	WRN28-3	WRN16-3	WRN16-3					
Accuracy (%)	69.42	59.90	64.04	69.50	68.76	70.37	70.71	71.63
GPU (s)	22.23	126.48 (PIs on CPU)	5.39 (GAFs on CPU)	15.03				
		+16.31 (model)	+16.31 (model)					
CPU (s)	29.63	126.48 (PIs on CPU)	5.39 (GAFs on CPU)	13.57				
		+29.94 (model)	+29.94 (model)					

## Data Availability

GENEActiv data was collected by the Exercise Science and Health Promotion Department of Arizona State University and is not currently approved for public release. PAMAP2 is public dataset that is available at https://archive.ics.uci.edu/dataset/231/pamap2%20638%20+physical+activity+monitoring. (accessed on 5 October 2025).
